# A Review of Non-Soil Biochar Applications

**DOI:** 10.3390/ma13020261

**Published:** 2020-01-07

**Authors:** Mattia Bartoli, Mauro Giorcelli, Pravin Jagdale, Massimo Rovere, Alberto Tagliaferro

**Affiliations:** 1 Department of Applied Science and Technology, Polytechnic of Turin, C.so Duca degli Abruzzi 24, 10129 Turin, Italy; mattia.bartoli@polito.it (M.B.); mauro.giorcelli@polito.it (M.G.); massimo.rovere@polito.it (M.R.); 2 Center for Sustainable Future, Italian Istitute of Technology, Via Livorno 60, 10144 Turin, Italy; pravin.jagdale@iit.it; 3 Faculty of Science, University of Ontario Institute of Technology, 2000 Simcoe Street North, Oshawa, ON L1G 0C5, Canada

**Keywords:** biochar, environmental remediation, energy storage, composites, carbon

## Abstract

Biochar is the solid residue that is recovered after the thermal cracking of biomasses in an oxygen-free atmosphere. Biochar has been used for many years as a soil amendment and in general soil applications. Nonetheless, biochar is far more than a mere soil amendment. In this review, we report all the non-soil applications of biochar including environmental remediation, energy storage, composites, and catalyst production. We provide a general overview of the recent uses of biochar in material science, thus presenting this cheap and waste-derived material as a high value-added and carbonaceous source.

## 1. Introduction

The use of carbonaceous material is one of the most established practices in material science [1]. Nowadays, carbon fibers are used in so many commodities that they have become an unavoidable asset for the global market [2]. Together with carbon fibers, carbon black leads the global carbon revenue due to its use in the production of tires [3]. Over the years, highly costly carbon materials such as carbon nanotubes and graphene have fed the dreams of the scientific community with their astonishing conductive, optical, and mechanical properties [4,5]. Despite the expected revolution, these materials did not develop from research and small productions. In 2009, Segal et al. [6] said that the world was ready for the ton-scale production of graphene, but, in 2020, single layer graphene is still sold at 230 €/cm^2^ and graphene oxide costs 140,000 €/kg [7]. In contrast, carbon black is sold for 1.5–0.95 €/kg [8]. High-tech carbon materials (i.e., carbon nanotubes, graphene and graphene oxide) have not yet fulfilled the promise of the new carbon era. While the world waits for the large scale commercialization of cheap pure carbon allotropes, new routes have been explored to make engineered carbon a profitable business. In this field, the most promising approach is probably the integration of carbon production with waste management [9,10,11]. The biomass waste stream is most abundant worldwide, and it is generally disposed through incineration. This represents both an environmental threat and an economic loss due to the transformation of a rich feedstock into heat and ashes. A more profitable approach is the thermal conversion of biomasses for the production of fuels [12,13], chemicals [14], and materials [15]. The conversion of biomass into liquid fuels is quite challenging due to the very high oxygen content of the original feedstock compared with traditional oil-derived fuel (i.e., gasoline and diesel). Nonetheless, the production of carbonaceous material from the thermochemical conversion of both lignocellulosic and non-lignocellulosic biomasses is very promising for several reasons. This bioderived carbon is generally known as biochar and could be used for many applications [16], mainly due to its properties and well-balanced cost of around 0.8–2.4 €/kg [17,18,19]. Currently, the most common biochar application is as for soil health improvement [20,21,22] and for use as solid fuels with a heating content of around 40 kJ/mol [23]. These two applications are limited and do not exploit the many biochar applications that can be derived from its easy tuneability with simple process adjustment [24].

In this review paper, we report a comprehensive overview of the non-soil applications of biochar to prove its feasibility as a replacement for traditional carbon materials and as a solid competitor with high tech materials.

We summarize the recent literature in four main sections dedicated to (i) environmental remediation, (ii) energy storage, (iii) composite production, and (iv) other applications.

We hope that this review is an useful tool to navigate the great sea of biochar potential.

## 2. Biochar Production Strategies

Biochar is produced through four main thermochemical routes: (i) torrefaction, (ii) pyrolysis, (iii) hydrothermal carbonization and (iv) gasification.

Torrefaction is a low temperature thermal treatment that is used to densify biomasses for energy purposes [25]. The operative temperature ranges from 200 to 350 °C with long residence and processing times and high solid product yields [26]. The carbon content of solid residue is around 50–60 wt.% [27] but can reach 72–80 wt.% by using microwave heating combined with the addition of microwave susceptors [28,29,30,31]. This approach leads to the reduction of the process timescale to minutes.

Pyrolysis is a high temperature thermal treatment that breaks polymeric macromolecules, thus giving compounds that have a lower molecular weight in an oxygen free atmosphere [12,32]. Pyrolysis is run with different heating technologies [33] and apparatus designs [34,35,36,37] at a temperature range from 450 to 700 °C [38] with huge variations in product fraction yields.

Hydrothermal carbonization is a thermal depolymerization process that is used to convert wet biomass into crude-like oil, gas, and hydrochar under moderate temperature and high pressure [39] by using an aqueous solvent [40], a non-aqueous solvent [41,42] or sub-critical/critical media [43]. This process can be performed with [44] or without a catalyst [42], and it is useful for the improvement of properties of products [45].

Gasification is the conversion of biomass into a gaseous fuel by heating in a gasification medium such as air [46], oxygen, or steam [47], generally at temperatures higher than 800 °C with or without a catalyst [48]. Products from gasification are a mixture of carbon monoxide, carbon dioxide, methane, hydrogen and water vapor. Biochar that is produced from gasification processes shows a higher carbon and ash content compared to biochar from torrefaction and pyrolysis processes [49]. This was due to higher process temperatures that promote an advance cracking process with the simultaneous reduction of volatile organic matters and an increment of fixed carbon.

**Figure 1:**
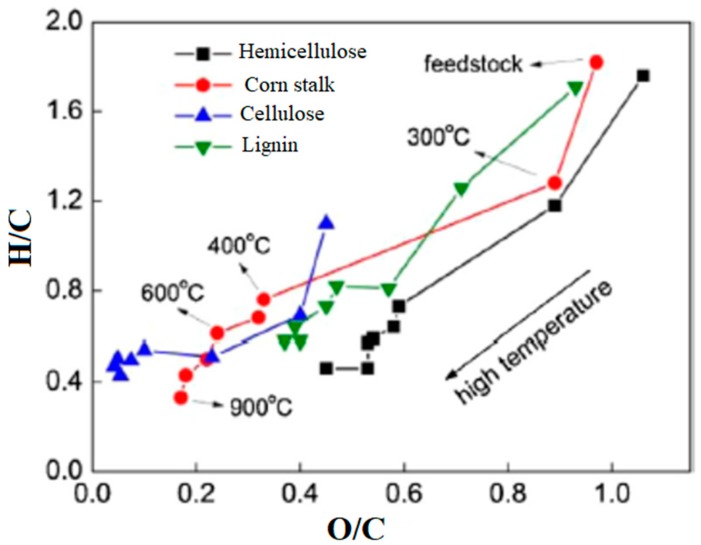
Van Krevelen diagram of corn stalk and corn stalk components during the thermochemical conversion process as reported by Gaojin et al. [50].

As shown in Figure 1, biochar carbon amount strongly correlates with the temperature that is adopted during the thermochemical conversion of all lignocellulosic biomasses.

Hydrogen/carbon and oxygen/carbon ratio low values are characteristic of less defective carbonaceous structures and could be more appealing for electronic and electric applications, while the other samples could be more useful as additive and for adsorbitive processes [51,52].

## 3. Non-Soil Biochar Applications

### 3.1. Environmental Remediation Applications

Environmental pollution is a global menace, and its magnitude is increasing day by day due to urbanization, heavy industrialization, and the changing lifestyles of people. In view of this, providing clean air, water and environments for people is a challenging task. In particular, the overall demand of water for human activities and the amount of wastewater that is produced are continuously increasing worldwide year by year [53]. Wastewater management has become one of the priorities for every urban conglomerate [54], involving several biological and chemical treatments [55] for reuse in civilian and industrial applications.

Water pollution is a global problem that is threatening the entire biosphere and affecting millions of lives [56]. Water pollution is recognized as one of the foremost global threats for human and environmental health [57]. A lot of different technologies for water purification are available based on filtration [58], adsorption [59], or degradation [60] technologies. Specialistic literature is rich in research that claims to have a water purification efficiency of up to 99%, but this is true only under idealized conditions of pH, contaminant concentrations, and other operating parameters [61]. Despite these astonishing claims, under real operative conditions, efficiency may substantially decrease. Furthermore, a lot of these techniques, such as ion exchange resins [62], are designed to target one class of contaminant at a time, which makes them useless in the case of environmentally polluted waters, where several contaminants simultaneously occur on a regular base. Taking into account these considerations, adsorption and degradative procedures are more appealing for real applications.

Biochar represents a game-changer material that is able to remove both inorganic and organic pollutants through adsorbitive and degradative processes. Furthermore, biochar could be successfully used for air purification by removing molecules such as carbon dioxide or hydrogen disulfide.

#### 3.1.1. Inorganic Pollutants Removal

Water pollution due to the presence of dissolved metal species has become a serious issue in a lot of underdeveloped [63,64,65,66] and developed countries [67,68]. The management of this issue is crucial for human health and safety.

Carbonaceous materials play a relevant role in the detoxification of watery sources, and biochar represents a very affordable solution. Huggins et al. [69] compared granular wood-derived biochar- and granular-activated carbon for the treatment of a wastewater stream in both batch and column systems. The authors clearly showed that biochar-based filtering material reduces the total chemical oxygen demand (COD) and ion concentrations (PO_4_^3−^, NH_4_^+^, As^3+^, Cd^2+^, Cr^3+^, Pb^2+^, Zn^2+^, and Cu^2+^) of wastewater treatment with more efficiency than activated carbon. Furthermore, biochar can be produced from so many feedstock sources that it guarantees a high versatility. Arán et al. [70] proved the relevance of feedstock by studying the copper adsorption of different feedstock-derived biochars. The authors clearly showed that the distribution of copper between the forms bound to biochar and dissolved into media depends on the starting material.

Chromium is a widely diffused element in the earth’s crust that has found a lot of applications. Consequently, chromium pollution has arisen as a serious environmental issue due to its abundant emissions from refractory materials, stainless steel production, and steel alloy production [71]. Chromium(VI) is particularly toxic, and its removal is mandatory to avoid both environmental and human life threats. Currently, many adsorptive systems are available [72,73,74], but they are quite expensive. Biochar has been used for the removal of Cr(VI) in a very effective way. Banerjee et al. [75] developed a zirconium-caged steam-activated biochar for the removal of Cr(VI) by using a contaminated water flux/fixed bed approach. The authors claimed a high Cr(VI) removal efficiency of up to 94 wt.% under a moderate flux rate (4 mL/min).

**Figure 2:**
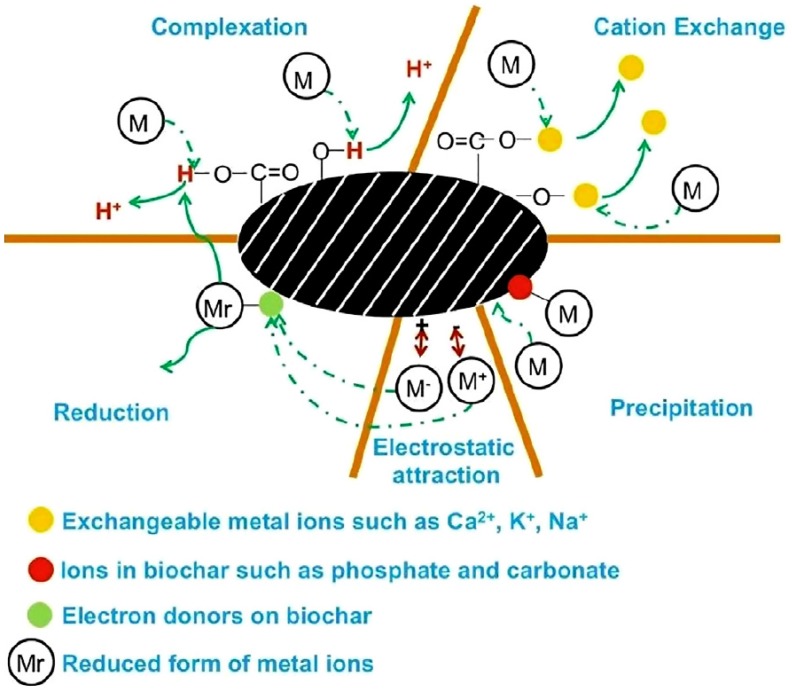
Mechanism of heavy metal adsorption onto neat biochar surface, as illustrated by Li et al. [82].

Another promising approach taken by various researchers is based on redox methodology that converts Cr(VI) into Cr(III) after adsorption onto a carbonaceous structure [76]. In one study, this route was combined with a proposed one-pot solvothermal method that was applied to synthesize an iron-decorated magnetic biochar composite [77]. Biochar acted simultaneously as an adsorbent for Cr(VI) and an electron-donor for the reduction of Cr(VI) to Cr(III), while iron-containing nanoparticles were involved in the immobilization of Cr(III). Iron-decorated biochar showed a remarkable ability to removal chromium of up to 84 wt.% with an easy recoverability. Similarly, Shi et al. [78] tailored iron nanoparticles to be supported on a mixed system that was based on silica and biochar. The authors showed how the tailoring process improved the Cr(VI) adsorption ability by up to 28 mg/g for a final removal of up to 85 wt.%. The essential role of biochar in Cr(VI) reduction in Fe–biochar systems has been proven by systematic studies [79,80], as shown in Figure 2, while in another study, the conversion of Cr(VI) to Cr(III) was ascribed to the redox couple Fe(III)/Fe(II) present in the greigite mineral that was used to tailor a biochar surface [81].

Adsorption onto neat biochar particles was also used to purify water streams from Cd(II), Pb(II), Cu(II), Zn(II), Sm(III) [83,84,85,86,87,88,89,90,91,92].

Biochar surface modification plays a very relevant role on the adsorptive performances of biochar-based materials.

One of the most established procedures to magnify functionalities on a biochar surface is partial oxidation during or after pyrolytic treatment. A low-oxygen pyrolysis atmosphere (1%–4%) was used by Zhang et al. [93] with a significant Pb(II) removal increment from 17.2 to 71.7 wt.%. This was confirmed by the study reported by Gao et al. [94], who used an oxidative post-pyrolysis procedure-based treatment with HNO_3_, H_2_O_2_ or KMnO_4_. This functionalization led to a 97.4 wt.% sorption of Pb(II) from the watery solution. Liatsou et al. [95] showed, through a detailed set of investigations of herbaceous biochars treated with HNO_3_, that anhydrides and carboxylic acids act as main surface groups to bind metal ions.

This assumption remains true for inorganic tailored biochar, as clearly showed by Feng et al. [96]. The authors described the effects of residual groups (i.e., hydroxyl, carbonyl, and carboxyl functionalities) on bromate removal when using a FeCl_3_-decorated biochar. This study clearly showed that the main removal mechanism of bromate was due to the oxidation of hydroxyl groups while an Fe(III)/Fe(II) redox couple served as electron shuttle to facilitate the electron transfer.

Biochar iron decoration represents a very interesting approach to produce a performing and highly recoverable adsorbent material with complex interactions between iron and carbonaceous phases [97]. Zhang et al. [98] impregnated an apple pomace biochar with a solution of Fe(III)/Fe(II) and used it with successful results in the adsorption of a watery metal mixture. A step forward is represented by the study of Zhou et al. [99]; the authors prepared a urea-functionalized Fe(O)-decorated biochar that guaranteed a high removal and recovery efficiency during the adsorption of Cu(II). Guo et al. [100] combined iron with manganese oxides, achieving the recovery performance of the iron-tailored biochars, together with the ability to adsorb both C(II) and As(V). Further biochar tailoring processes have involved the introduction of phosphate residues that create a micropores structure together with a high surface area [101], as well as simple and complex organic [102,103] and inorganic [104,105] frameworks.

Anionic species that are dissolved in water represent another great family of watery pollutants; however, in this case, biochars still represent a valuable tool [106,107,108,109].

Phosphates are probably one of the principal causes of the eutrophication of surface waters [110,111,112,113]. Trazzi et al. [114] reported the use of a Miscanthus biochar produced at 700 °C for removing phosphates and segregating them into the soil, thus improving their agronomic performances and reducing algae proliferation.

Several biochar modifications have been used to tailor phosphorous uptake, ranging from electrochemical [115] to inorganic deposition [116,117] procedures. These studies led to the development of real-scale plants based on biochar adsorbents that operate in flux and not merely on the batch scale [118].

Nitrate represents another anion species that is strongly correlated with eutrophication [119]. Divband et al. [120] developed a biochar from the pyrolysis of sugarcane bagasse, showing the best operative conditions after 1 h of using a solution with a pH of 4.64 and a starting nitrate concentration of up-to-a-dose-adsorbent of 2 g/L. Furthermore, species such as fluoride [121] and uranyl oxides [122,123] could be efficiently removed from watery phases by using biochar.

Biochar destiny after adsorption represents another strong point for its use for water purification. As reported by several authors [124,125], contaminated biochar could be a source of fertilizers, catalysis, metal nanoparticle synthesis through pyrolytic conversions, feed additives, and biologically active compounds.

The desalinization process represents a further relevant application of water treatments that use biochar. This procedure has been performed through simple osmotic filtration [126] and through capacitive processes [127] with [128,129] or without [130] functionalization.

#### 3.1.2. Organic Pollutants Removal

Watery pollution, due to the presence of organic molecules, has risen together with the anthropization. The anthropogenic effect is the main cause of the release of pollutants such as dyes, pharmaceuticals, and polymers residues [131,132]. During last few decades, the use of carbonaceous materials for the improvement of water purifications has been very intensively studied. Materials such as carbon dots [133], carbon nanotubes [134] and graphene [135] have been used for both organic pollutants detection and removal. Despite their performances, the high cost of high-tech carbon materials has slowed down their application in real-plant units.

**Figure 3:**
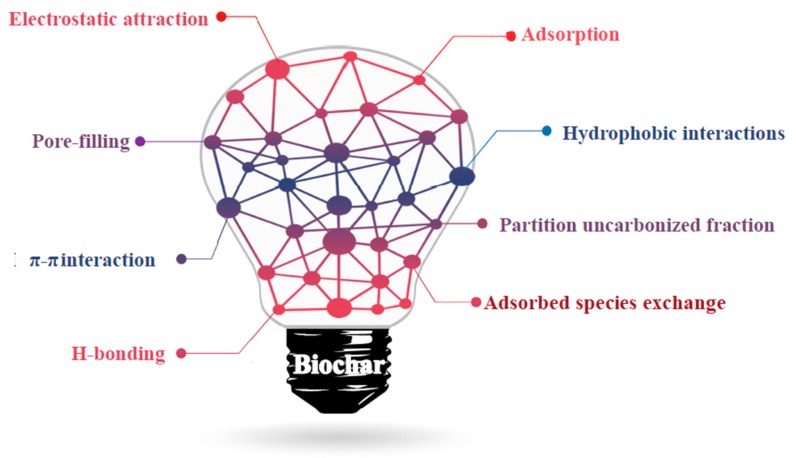
Schematic interactions occurring between organic molecules and biochar particles, as reported by Dai et al. [144].

Biochar represents a cheaper solution compared to other carbonaceous materials with very promising performances [136,137] due to the several interactions that occur on biochar particles, as summarized in Figure 3.

The great variety of interactions that occur between biochar and organic molecules ranges from very weak (e.g., hydrophobic ones) to very strong (e.g., hydrogen bond and π–π orbital interactions).

The simultaneous occurrence of these interactions is the reason for the good performance of biochar as an adsorber for several typologies of compounds [138,139,140,141,142,143].

Firstly, biochar has been used to remove persistent small organic molecules such as aromatics. Jayawardhana et al. [145] used biochar that was derived from the pyrolysis of municipal solid waste to remove alkylated benzenes. They reached an efficiency of 850 and 550 μg/g for toluene and m-xylene, respectively. Similarly, Kang et al. [146] used a biochar-derived material for the adsorption of phenanthrene. Nitroaromatics were also removed by using rice husk biochars, as described by Lingamdinne et al. [147]. In this research, the authors used a rice husk that had been pyrolyzed at 700 °C to remove 2,4,6-trinitrotoluene and 1,3,5-trinitro-1,3,5-triazacyclohexane. They showed that the adsorption process occurred through weak electrostatic interactions as well as through charge transfer between nitric functionalities and biochar surface functional groups. Mandal et al. [148] used various biomass wastes (tea, cucumber, and mixed hardwood) for the production of biochar at 400 and 700 °C. These carbonaceous materials were used to adsorb 2,4-dichlorophenoxy acetic acid, achieving an uptake of up to 59 mg/g. Zhu et al. [149] deeply investigated the interaction between biochar and 2,4-dichlorophenoxyacetic acid. They showed that surface amination and oxidation can improve biochar adsorption properties. Furthermore, polyhalogenated hydrocarbons were removed from waste stream by using biochar that was produced from the pyrolysis of digestate with an uptake of up to 11 mg/g [150].

Al Ameri et al. [151] used a peat-derived biochar as a bio-sorbent for the sorption and removal of crude oil spills from synthetic seawater, reaching a crude oil adsorption of 32.5 g per gram of biochar sorbent. Similarly, Feng et al. used porous carbon macro spheres with a diameter of 1–2 cm that were prepared through the carbonization of the fruit of *Liquidambar formosana*. The authors claimed an oil adsorption close to 99 wt.%.

Dyes represent the other great threat to water sanification due to their persistency and toxicity [152]. The adsorption of dyes is affected by many parameters, such as solution pH, chemical nature and initial concentration of the dye molecules [153]. He et al. [154] reported the use of a micro-scale biochar particles/polysulfone mixed matrix hollow fiber membrane for the removal of methylene blue from water. The membrane’s static and dynamic adsorption performance was investigated, and the adsorption mechanism was associated with electrostatic interaction, hydrogen bonding and hydrophobic interaction. In a study by Hou et al. [155], hydrochars from bamboo shoot shells were used for rhodamine B adsorption. Hydrochar produced at 800 °C with a heating rate of 25 °C adsorbed up to 86 mg/g of rhodamine B—a result that was lower than the results that were achieved by using non pyrolyzed sugar cane bagasse [156]. Zazycki et al. [157] used pecan nutshell biochar as low-cost adsorbent for removing Reactive Red 141 from aqueous solutions with an uptake of up to 130 mg/g, which was comparable with results achieved by Netpradit et al. [158], who used metal hydroxides. Jung et al. [159] produced a magnetic iron-decorated biochar from the pyrolysis of marine macroalgae for the adsorption of Orange 7 from aqueous media with comparable performances to those of metal frameworks [160]. The iron tailoring enabled a higher adsorption performance along with an easier separation and recovery process in the post-adsorption stage when using a simple magnet. A similar approach was applied by Heo et al. [161], who used a CuZnFe_2_O_4_-tailored biochar composite for the simultaneous removal of bisphenol A and sulfamethoxazole.

Another rising issue in civilian water is the presence of traces of pharmaceuticals compounds due to their consumption [162,163] and inappropriate disposal [164]. Kim et al. [165] proposed an interesting ultrafiltration-activated biochar hybrid system for the removal of ibuprofen, 17 α-ethinyl estradiol, and carbamazepine, ultimately achieving an adsorption of up to 47 wt.%. Li et al. [166] used biochars that were prepared from cassava dregs at different pyrolytic temperatures (350, 450, and 700 °C) for the adsorption of ciprofloxacin, and this process is correlated to the action of residual groups with adsorption ability. Jang et al. [167] used a sodium hydroxide-activated biochar to remove tetracycline; a comparison with a commercial activated carbon (Calgon F400) showed a comparable activity with the biochar used. Similarly, Mandal et al. [168] removed atrazine and imidacloprid from water by using an agricultural waste stream-derived biochar in a multi-staged batch adsorption systems. A different approach was reported by Xu and co-workers. They used Fe(0) nanoparticle-tailored biochar for the adsorption of florfenicol [169]. An additional sulfide modification of the iron nanoparticles led to the disruption of the antibiotic molecules.

**Figure 4:**
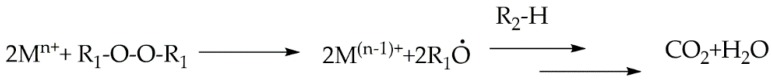
Schematic processes of the organic molecule degradation mediated by peroxides.

Adsorption processes are neither the only nor the most used route to eliminate organic pollutants from watery streams. Degradative processes based on the oxidation routes play a major role in this field, mainly through catalytic-mediated peroxide oxidation, as shown in Figure 4.

Fenton and Fenton-like processes are the more effective processes based on the activation of peroxides in mild conditions by using cheap metal precursors [170] and more stable oxidant agents such as persulfates [171]. Huang et al. [172] proved the significant role of biomass types on the formation of persistent free radicals during Fenton and Fenton-like process. This was particularly interesting because biochars have shown high catalytic potential due to their persistent free radicals that attract attention in the removal of refractory pollutants from water. The radical evolution in biochars that were derived from several biomasses (i.e., bamboo, corn stalk, and pig manure) were investigated by electron paramagnetic resonance. These experiments, together with linear sweep voltammetry measurements, showed that a hydroxyl radical was the dominant reactive radical in the biochar–H_2_O_2_ systems. Based on this study, He et al. [173] described persulfate activation with sawdust biochar in an aqueous solution with an enhanced electron donor-transfer effect. The authors pyrolyzed sawdust at two different temperatures (300 and 700 °C), showing that the degradation efficiency of Orange 7 increased along with the pyrolytic temperature. This was due to the graphite electron donor-transfer complex formed on the surface and in the pores of the biochar that played a decisive role in the reaction.

Interestingly, Ho et al. [174] produced a nitrogen-doped biochar from the pyrolysis of C-phycocyanin extracted spirulina residue for catalytic persulfate activation. The authors processed the feedstock at a high temperature (900 °C) and achieved nitrogen doping directly from the protein content. The resulting material promoted a non-radical activation process that guaranteed a mild and high-efficiency strategy for disinfection in waste and drinking water. More traditional catalysts that produced a porous biochar originated from *Myriophyllum aquaticum* tailored with Fe_3_O_4_ were described by Fu et al. [175]. This catalyst induced the activation of peroxymonosulfate during p-hydroxybenzoic acid degradation according to the traditional Fenton process route. Similarly, several authors have claimed the effectiveness of magnetic iron-based biochars in Fenton degradative processes [176,177]. Furthermore, Deng et al. described the use of pyrolyzed wood-waste for the fabrication of a porous carbon cathode that acts in the electro-Fenton degradative process of sulfathiazole in a pyrophosphate electrolyte alkaline environment. Bisphenol A could be also degraded by using both thermal [178] and ultrasound [179,180] induced Fenton processes.

Gan et al. [181] induced the degradation of refractive organic pollutants, such as dimethyl phthalate, by using a metal framework based on CoFe_2_O_4_ for the activation of peroxymonosulfate.

Fenton and Fenton-like routes are not the only available oxidative procedures. Moussavi et al. [182] prepared a biochar from a pistachio hull that demonstrated its catalytic potential for degrading Reactive Red 198 in catalytic ozonation processes, ultimately achieving a 58 wt.% removal efficiency.

Alternatively to oxidative degradation processes, reductive processes can also be performed, even if they are less appealing than the others due to their greater complexity. Some authors have described a reductive approach for the removal of nitro alkylated benzene based on the use of Fe(0)-tailored biochars [183,184], but the number of studies is still much lower than those on oxidative-based processes. A different and interesting approach is the hybrid system proposed by Lyu et al. [185] based on the use of biochar-supported nanoscale iron sulfide and Corynebacterium variabile HRJ4. The authors applied this chemo-bio route for the dechlorination of trichloroethylene. A similar approach was used by Ayyappan et al. [186], who used a coconut shell biochar for dye degradation in a microbial fuel cell; they claimed a removal efficiency of up to 78 wt.%.

Photodegradative procedures have also been considered for watery streams purification [187]. Shirvanimoghadda et al. [188] produced and used carbon microtubes from the pyrolysis of cotton waste at different temperatures (from 900 to 1500 °C) for the UV photodegradation of bisphenol-A. A tailored magnetic biochar-containing Fe_3_O_4_-BiOBr was used by Li et al. [189] for carbamazepine photodegradation under visible LED light irradiation with a degradative performance of up to 96 wt.%. Kumar et al. [190] described another bismuth-based magnetic biochar material that was successfully used for the UV photodegradation of paraquat, with the nitrophenol reduction achieving a degradation of up to 99 wt.%. These performances were quite comparable with more expensive materials such as tailored graphene oxide [191]. Furthermore, the combination with cheap photoactive species such as zinc oxides has led to the realization of a performing photoactive material [192].

#### 3.1.3. Gaseous Pollutants Removal

Gas mixture purification is one of the most relevant industrial issues [193]. Actually, the most used approaches are based on selective membranes [194,195] or in-solution absorption [196]. The use of a biochar-based adsorber could be an interesting application on a large scale for this bio waste-derived material. Das et al. [197] realized a biochar packed biofilter for gas-phase hydrogen sulfide removal; the authors claimed a very good stability for fifty days of operation. This system showed a maximum elimination capacity of 33 g/m^3^ h, along with a fast response to shock loads. A very similar approach was described by Braghiroli et al. [198] for the removal of SO_2_ that was generated from anthropogenic sources. Shao et al. [199] used activated biochar, proving the beneficial effect of CO_2_ activation in producing a material with a higher adsorption capability and a higher regenerability compared with pristine biochar.

Furthermore, the use of carbon dioxide as a biochar activation reagent could contribute to the reduction of CO_2_ atmospheric emissions, which represent one of the greatest threat for climate change [200,201,202]. Nonetheless, the use of CO_2_ for biochar activation is not the only route to mitigate emissions. The other and more appealing approach is represented by the use of biochar for the removal of CO_2_ from gaseous mixtures [203]. Liu et al. [204] described the use of spent coffee grounds as efficient CO_2_ adsorbers, reaching a gas uptake of up to 119 mg/g at 35 °C. Igalavithana et al. [205] recovered biochar from the gasification of food and wood waste, ultimately claiming a high CO_2_ uptake and a very good recyclability. Huang et al. [206] used a biochar that was produced from the microwave co-torrefaction of sewage sludge and *Leucaena* wood, ultimately reaching a CO_2_ uptake of up to 53 mg/g. Chiag et al. [207] explained different biochar CO_2_ adsorption abilities with the surface microstructures and residual functionalities of carbonaceous materials. The effect of nitrogen residual functionalities was used by Zhang et al. [208] for the realization of a nitrogen-rich rice husk biochar that was able to adsorb CO_2_ at a rate of 59 mg/g. Rice husk was also pyrolyzed under microwave irradiation [209] and activated with post-pyrolysis treatments [210], ultimately showing very promising CO_2_ uptake values. Pyrolysis post-treatments were widely used to increase the CO_2_ adsorption ability of a biochar by introducing basic sites via ammonia functionalization processes [211,212].

### 3.2. Energy Storage Applications

**Figure 5:**
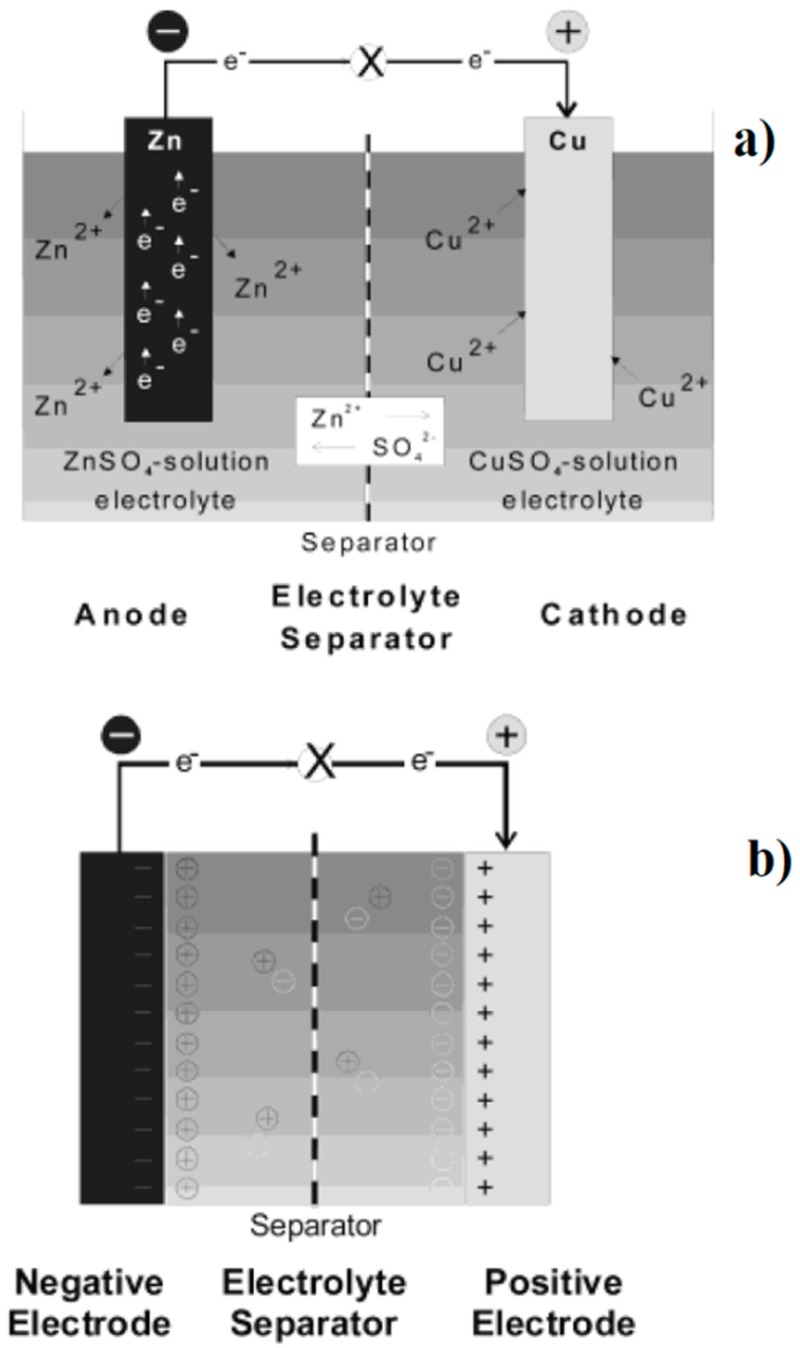

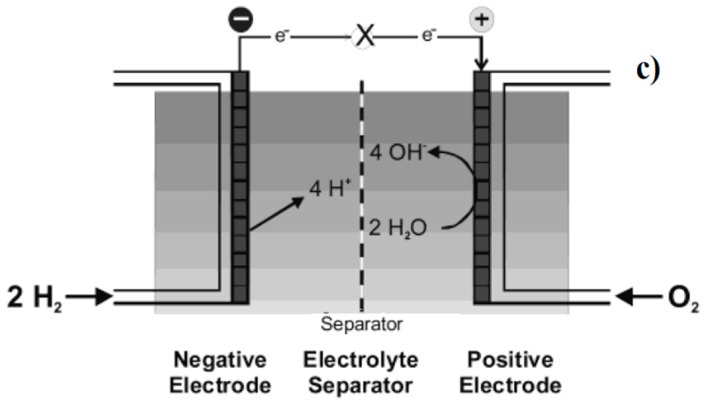
Schematic representation of (**a**) battery (Daniell cell), (**b**) a supercapacitor, and (**c**) a hydrogen fuel cell as reported by Winter et al. [219].

Energy storage technology represents a great challenge of 21st century [213] due to its different applications. Nowadays, numerous technologies [214] have been developed, such as solar and fuel cells [215,216], high performance batteries [217] and supercapacitors [218], as summarized in Figure 5.

A battery is a system formed by at least two electrochemical cells with contacts to supply electrical energy according to electrochemical potential. Specialist literature has been focused on two main solid state battery systems based on lithium [220] and sodium [221] ions. A supercapacitor is an energy storage modulus that stores energy in an electrical double layer that is formed at the interface between an electrolytic solution and an electronic conductor. A fuel cell is an electrochemical system that produces electric energy through the supply of a fuel (i.e., hydrogen [222], carbon [223], and methanol [224]) and an oxidant agent (i.e., oxygen and hydrogen peroxide).

#### 3.2.1. Biochar Used for Supercapacitor Production

The essential requirement for producing a performing supercapacitor material is an elevated surface area where the double ionic layer can be created. For this purpose, physically- and chemically-activated biochar is a very attractive material for the realization of supercapacitor electrodes [225]. Chemical activation introduces functional groups on the surface of an activated biochar, thus affecting the latter’s electrochemical properties [226]. Nonetheless, Gabhi et al. [227] described the effect of monolithic biochars with graphite and graphite-like structures on capacitive performance. The authors showed the relationships between conductivity and activate biochar structures at 950 °C by using sugar maple, oak and hickory woods. They claimed an increase of biochar conductivity from 5 × 10^−6^ up to 343 S/m when carbon content changed from 86.8 to 93.7 wt.%. This phenomenon was attributed to the formation of graphite nanocrystals in the main structure of the biochar during the high temperature treatment.

Chemical activation is a well-established procedure to create an activated biochar with a good capacitive performance. Luo et al. [228] reported cellulose activation by using ammonia, and they were able reach area capacitance of 40 mF/m^2^.

Jin et al. [226] described the chemical activation of an ash-rich biochar by using potassium hydroxide at 900 °C, with a further modification occurring when using HNO_3_ at 150 °C. Activated biochar showed a very high specific area of up to 2000 m^2^/g with a specific capacitance of up to 260 F/g.

A low ash content feedstock was used by Qu et al. [229] for a direct conversion into activated biochar by using a steam and acidic–alkali treatment. Corncob-activated biochar had a surface area of up to 1210 m^2^/g, a capacitance of 314 F/g, and a remarkable stability after 10^5^ cycles in a symmetrical cell. Fast pyrolysis and alkaline chemical activation was used by Chen et al. [230] for the conversion of rotten food waste into an activated biochar with a capacitance of 488 F/g. Herbaceous feedstocks were diffusely studied for electrodes production and feedstock like hemp [231] and several flowers [232,233] have been used for supercapacitor realization.

Surface morphology plays a relevant role in biochar-based supercapacitors [234,235,236]. As a matter of fact, macroporous biochar is characterized by an inferior performance compared to micro and mesoporous biochar, while microporous biochar has been shown to work at higher current density—up to 1.3 A/g—compared to other forms [237].

Activated biochar properties could be also modulated by using a plasma treatment, as described by Gupta et al. [238]. The authors reported a low temperature oxygen plasma treatment that was able to magnify the capacitance of a yellow pine biochar from 14 to 174 F/g as consequence of a surface area significant increment.

Furthermore, non-lignocellulosic biomasses could be converted into usefully carbonaceous materials for capacitive uses. As an example, keratin-mixed algae [239] was pyrolyzed for the production of heteroatom-doped activated biochar with interesting surface area and capacitive values. Pontiroli et al. [240] reported the production of a hierarchically-porous activated biochar from the pyrolysis of poultry litter with specific surface area of up to 3000 m^2^/g and a capacitance of 229 F/g.

#### 3.2.2. Biochar Used for Batteries Production

Several authors have explored the use of biochars as anodic materials for the realization of performing batteries. Many authors have focused on the realization of lithium ion batteries due the great demand of highly technological devices based on them. Dai et al. [241] produced biochar from the pyrolysis of sewage sludge in order to produce hierarchical porous hollow carbon nanospheres with a great surface area of up to 1500 m^2^/g. This biochar was employed as an anode for an Li-ion battery and showed an impressive discharge capacity of up to 1169 mAh/g. Low porousity biochars have shown far lower performances, as reported by Luna-Lama et al. [242], who used spent coffee grounds pyrolyzed at 800 °C and reached a specific capacity of only 360 mAh/g. This trend was confirmed by Zhang et al. [243], who reported values of around 600 mAh/g when using a microporous biochar. Similarly, Benitez et al. [244] reported the use of microporous biochar as a cathodic material for a lithium–sulfur battery with a specific capacity of 915 mAh/g and a current density of 100 mA/g. Chen et al. [245] showed that nitrogen doping could enhance hierarchical porous biochar activity derived from the pyrolysis of derived pomegranate residues at 700 °C of up to 550 mAh/g. Furthermore, the nitrogen-doped material was particularly stable after 500 cycles thanks to the chemical confinement of sulfur and the soluble lithium polysulfides performed by nitrogen sites. Heteroatom-doped biochar was used by Chen et al. [246] as a cathode with a discharge capacity of up to 1049 mAh/g. Non-lignocellulosic biomasses were used, as reported by Magnacca et al. [247]—the authors used chitin pyrolyzed at a moderate temperature for the realization of a low cost lithium–sulfur battery with acceptable performances. The tailoring process could further enhance biochar performance. Pan et al. [248] decorated a silk-derived biochar with nanocubes of ZnCo_2_O_4_ to produce a flexible performing anode material. Similarly, Li et al. [249] tailored a pomelo pericarp biochar with Fe_3_O_4_ nanoparticles, reaching a capacity of up to 635 mAh/g. Salimi et al. [250] combined the Fe_3_O_4_ nanoparticle-tailoring process with the pyrolysis of algae to produce an electrode material with a higher initial specific discharge capacity of up to 740 mAh/g and a good cyclic stability [251].

Different ion-based batteries have also been developed, though in minor quantities; the only solid works about is from Saavedra Rios et al. [252], who used biochars from various biomasses as precursors for hard carbon anodes in sodium-ion battery applications.

#### 3.2.3. Biochar Used for Fuel Cell Production

Several authors [253,254,255] have used biochar as a fuel for direct carbon fuel cells, proving the relationship between biochar properties (e.g., carbon percentage, ash content, surface area, and heating value) and its fuel performances. Xu et al. [256] definitively established the direct relation between the thermal degradation of biomasses and their performances in carbon fuels cell by comparing thermogravimetric analysis with empirical data. Qiu et al. [257] reported the development of a direct carbon solid oxide fuel cell that was able to convert the chemical energy of biochar into electricity with high efficiency. The authors used biochar that had been derived from several biomasses (wheat straw, corncob, and bagasse), reaching a peak power densities of 260 mW/cm^2^ at 800 °C. A detailed study of direct carbon fuel cells was reported by Kacprzak et al. [258,259,260]. Firstly, the authors compared graphite rod and biochar from an apple tree that was pyrolyzed at 600 °C in a molten salt mixture of sodium and potassium hydroxide, and they found an optimum operative condition at 400 °C with a NaOH/KOH ratio of 1. In these operative conditions, biochar outputs were comparable with those achieved when using pure graphite. A further experiment proved the similar behavior of biochar and commercial coal with a generated power, in both cases, close to 35 mW/cm^2^.

In the same field, Ali et al. [261] used titanate-based anodes in a direct carbon fuel cell by using biochar from pyrolyzed walnut and almond shells as fuel. The authors claimed a generated power of up to 78 mW/cm^−2^. Elleuch et al. [262]—in similar conditions without the titanate-based anodes—reached a power of up to 127 mW/cm^2^.

Another appealing use of biochar is the realization of electrodes for microbial fuel cells, as reported by Huggins et al. [263]. The authors used wood-based biochars as microbial fuel cell electrodes to significantly reduce costs and carbon footprints, showing a generated power of 532 ± 18 mW/m^2^, with power cost of power output cost 17 $/W. This was 90% cheaper than graphene-based fuel cell electrodes, which have a cost of up to 402 $/W. Further improvements were achieved by using a manganese oxide-doped biochar, thus improving the power output by up to 606 mW/m^2^ [264]. Khudzari et al. [265] developed a granular biochar anode in rice plant microbial fuel cells that were focused on the production of bioelectricity; the authors showed the beneficial effect of biochar on reducing methane emissions without decreasing plant biomass yield.

Biochar was also used for the production of performing cathode electrodes. Li et al. [266] produced biochar from the pyrolysis of corncob (with the temperature ranging from the torrefaction range up to 750 °C) that was used as an oxygen reduction reaction catalyst in air cathode microbial fuel cells; here, the biochar produced at 650 °C showed higher power outputs of up to 459 mW/m^2^. Similarly, Yuan et al. [267] used a sewage sludge biochar produced at 900 °C to reach power outputs of up to 500 ± 17 mW/m^2^.

Apart from the electrodes, Chakraborty et al. [268] developed a novel, low-cost proton exchange membrane that used sulfonated biochar that was produced from rotten food that had been pyrolyzed at 600 °C for application in microbial fuel cells. This study proved the high performances of the membrane, with a proton conductivity of 0.07 S/cm, an ion transport number of 0.891, and an oxygen diffusion coefficient pf 6.5 × 10^−9^ m^2^/s. Comparing proton conductivity and power harvested per unit, the biochar-based membrane outperformed those based on materials such as Nafion.

### 3.3. Biochar-Based Composites Production and Properties

**Figure 6:**
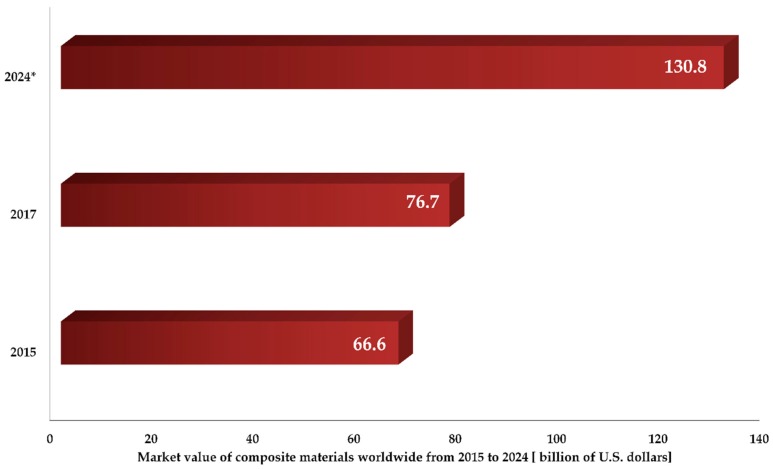
Composite material global market revenue, with a prediction for 2024 as reported by Grand Vie Research Center [269].

Nowadays, composite materials represent one of the largest global markets, with an expected future development of up to 131 billion dollars in 2024, as shown in Figure 6.

**Figure 7:**
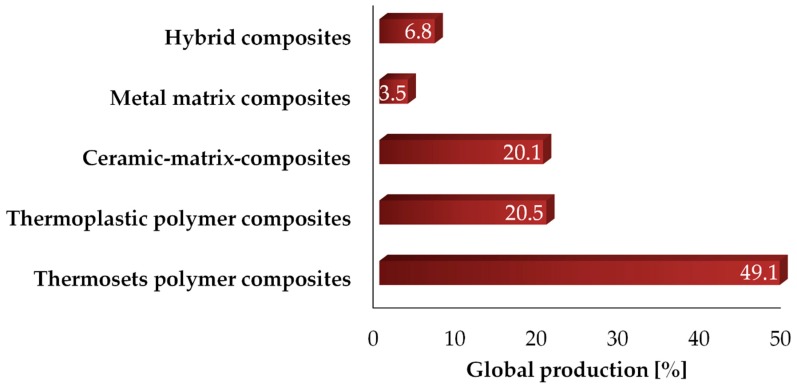
World carbon-based composite production in 2018, according to Sauer et al. [270].

Carbon-based composites represent one of the most relevant parts of global markets, with an annual production of about 150 kton/y in 2018 [270]. As shown in Figure 7, around 70% of total carbon-based composites are represented by polymeric host materials, with 49.1% being thermoset and 29.5% being thermoplastic polymers. Among them, carbon fiber-reinforced epoxy resins represent a greater amount due to their many applications in key high-tech sectors such as aeronautics and aerospace industries [271]. Carbon-containing inorganic composites are mainly represented by ceramics [272] and cements [273], but their total production is far lower than polymers. In this scenario, biochar plays a minor role, even if its use is going to be consolidated due to production flexibility and its property tuneability [274].

#### 3.3.1. Biochar–Inorganic-Based Composites

Cement production is a one of the largest productions in the world, with more than 3 Gton/y produced in 2018 [275]. Along history, many additives have been developed to enhance both the mechanical properties and durability of cement-based material [276], ranging from polymers [277] to carbonaceous materials such as carbon nanotubes [278], graphene [279] and carbon fibers [280]. Biochar has also been extensively studied, even if it has not yet reached the market.

Cosentino et al. [281] evaluated the performance of a standardized biochar set that was produced by UK Biochar Research Center [282], considering flexural strength and fracture energy. The authors reported inferior performances compared to those achieved by previous studies [283]. Nonetheless, they reported a comprehensive study about the influence of a solid set of biochar properties (e.g., carbon content, pyrolysis temperature, and particle size) on the mechanical properties of biochar-containing cement composites. A further insight into biochar-based cement composites was reported by Gupta et al. [284]. The authors reported an exhaustive study on the influence of biochar particle size and surface morphology on the rheology, strength and permeability of cement mortar under both moist and dry curing conditions. The authors showed the primary effect of biochar macroporosity of big sized particles (diameters ranging from 2 to 100 µm) on the rheological properties of cement mortar. Biochar particle size did not affect the hydration process, which was fast in all experiments run. Small sized particles (diameters below 2 µm) improved early strength and water tightness compared to big size macroporous biochar particles. Mo et al. [285] combined biochar and MgO to mitigate the autogenous shrinkage of cement materials, and similar results were achieved by Muthukrishnan et al. [286] by simply using low-ash pyrolyzed rice husks. Gupta et al. [287] explored the addition of pre-soaked biochar particles that were produced at 500 °C, and they showed a reduction of sorptivity and a depth of water penetration of up to 60%. Another interesting matrix that is able to host biochar is concrete. Concrete is more complex compared to neat cement, and it also contains inert material such as the sand of fine milled stones. The content of cement is highly variable and could partially or totally be replaced by biochar, as reported by Dixit et al. [288]. The authors described the use of biochar as a material for cement replacement in ultra-high performance concrete. The authors firstly described the biochar–concrete interphase interactions by using scanning electron microscopy to enlighten the deposition of cement hydrates on the surface and inside the surface pores of biochar, with dense interfacial transition zone, further suggesting the efficacy of biochar for improving hydration.

Gupta et al. [289] also proved that the addition of biochar that had been pyrolyzed at 550 °C improved concrete elevated temperature properties far better than fume silica, with a strength increment of up to 20%. Biochar concrete composites showed interesting properties for the sound adsorption across the range of 200–2000 Hz, as reported by several research papers [290,291]. One of the most promising discoveries was comprised of the outputs presented by Kua et al. [292]. The authors described the use of biochar-immobilized bacteria mixed with poly(vinylalcohol) fibers for the production of a self-healing fiber-reinforced concrete. The authors claimed the ability of self-repairing cracks greater than 600 µm.

Cement and concrete are not the only inorganic matrixes that have been used to host biochar. Mu et al. [293] deeply described the use of carbon-containing clay composites as building materials. In this field, biochar has found many applications in construction science. Lee et al. [294] produced a hybrid material based on biochar and natural clay used as building envelope insulation with an increment to water vapor resistance due to the presence of up to 23 wt.% of biochar. The mechanical properties of biochar-based clay composites were described by Yang et al. [295]. The authors tested biochar that had been produced from several biomasses (e.g., rice husk, coconut shell, and bamboo) and showed an improvement in thermal performance and strength from a 10 wt.% mixture of bamboo-derived biochar and red clays.

Dahal et al. [296] used biochar as a filler in glass–fiber composites and showed lower damping ratio, an elevated storage moduli of up to 4 GPa, and a higher stiffness for the 10 wt.% biochar composite, as compared to the neat glass fibers.

#### 3.3.2. Biochar-Containing Reinforced Plastics

Carbonaceous-reinforced thermoset plastics are widely diffuse materials that incorporate a plethora of different matrixes [297,298,299]. An epoxy matrix is the most studied and the most used matrix worldwide. Consequently, the replacement for traditional carbon fillers with biochar has stimulated great interest. Khan et al. [300] studied the mechanical and di-electrical properties of high-temperature annealed maple biochar-based epoxy composites by using a filler concentration ranging from 0.5 to 20 wt.%. The authors claimed a magnification of all mechanical properties when using an annealed biochar load of up to 4 wt.% and similar dielectric properties of low-loaded carbon nanotubes resin when using 20 wt.% of biochar. Bartoli et al. [301] established the relationship between the surface morphology of biochar particles and related composite mechanical properties by using a biochar loading of 2 wt.%. The authors achieved a 40% increment of maximum elongation when using a rhizomatous grass biochar and doubled the Young’s modulus when using a wheat straw-derived biochar. The authors advanced the hypothesis that a smooth surface can induce an improved mobility inside the epoxy matrix, while a highly porous surfaces could not. This was reflected in the different behaviors that were observed when using biochar that was produced in the same pyrolytic conditions as different feedstocks.

Pyrolytic temperature plays a crucial role in the interactions between epoxy resins and biochar particles. Giorcelli et al. [302] used a maple tree-derived biochar that was produced at 600 and 1000 °C, and they observed a maximum elongation improvement of up to five times compared with neat resin.

High temperature-treated biochar could be a solid choice for the production of conductive epoxy composites. Giorcelli et al. [303] reported that more graphitized biochar showed a strong DC electrical conductivity. This affected the ability of these materials to shield the microwave radiation with a comparable performance to multiwalled carbon nanotubes [304], even as thin films [305].

Furthermore, biochar from pyrolyzed, wasted cotton fibers could be recovered in a carbon fiber shape that showed the property enhancement of an epoxy resin host matrix [306,307].

Regarding thermoplastic-reinforced plastics, polyolefins-based biochars are the most produced. Among them, biochar-containing polyethylene was studied by Arrigo et al. [308] by using a coffee-derived biochar. The authors reported that the rheological and thermal behavior of biochar composites showed a slowing down of the dynamics of the polymer host matrix due to the confinement of the polymer chains on the filler porous surface. Additionally, the well-embedded biochar particles improved the thermo-oxidative stability of the produced polyethylene composites. Zhang et al. [309] studied the influence of temperature on poplar biochar-based high density polyethylene composites. Interestingly, the microcrystalline structure of the polymer matrix was not affected by the presence of biochar. On the other hand, the mechanical properties showed an appreciable difference between neat and biochar-loaded polymers, with the latter showing an improved flexural strength and a decreased impact strength. Li et al. [310] studied the behavior of highly biochar-loaded, ultra-high molecular weight polyethylene. The authors realized a performing electromagnetic interference shielding material by using 80 wt.% of bamboo biochar that was pyrolyzed at 1100 °C and reached a conductivity of 107.6 S/m. Furthermore, Bajwa et al. [311] reported the utilization of biochar for the production of a high density polyethylene/poly(lactic acid)/wood flour composites with super thermal stability properties.

Poly(propylene) is the other widely studied polyolefin for the realization of biochar-based composites. Das et al. [312] proved the economic feasibility of the use of biochar over traditional carbon fillers. The authors showed the cost reduction of biochar-based composites with the same properties of non-biochar-based ones due to the reduction of compatibilizer amounts of up to 3 wt.%, with 18 wt. % of saving. The low cost of biochar was the key of the study of Behazin et al. [313], where the authors used a pyrolyzed perennial for the realization of a poly(propylene)/poly(octene-ethylene) composite with filler loadings of 10 and 20 wt.%; they showed, through rheological analysis, stronger interactions between the polymer matrix and biochar. A detailed study of poly(propylene) and biochar interaction was reported by Bhattacharyya and co-workers in several papers [314,315,316]. During this research, the authors proved the general improvement of the mechanical and thermal properties of biochar-based poly(propylene) composites together with a significant effect in flame retardancy. Furthermore, Das et al. [317] produced biochar-based wood polymer composites while manufacturing a biocomposite with appreciable properties. Similarly, Poulose et al. [318] combined date palm biochar with a poly(propylene) matrix and revealed that the biochar had negligible effect on the storage modulus up to a 15 wt.% loading. Other widely used polyolefin matrix used for the realization of piezo sensors are poly(vinyl alcohol) [319,320] and poly(acrylonitrile) [321].

Furthermore, polyesters were impregnated with biochar in a study by Ogunsona et al. [322]. The authors filled nylon 6 with biochar produced from the pyrolysis of *Miscanthus* at 500 and 900 °C, and they showed the beneficial effect of the high temperature-produced biochar and the detrimental effect of the other. Sheng et al. [323] modified bamboo biochars with silyl groups for the production of poly(lactic acid) composites, showing an enhancement of maximum elongation of up to 93% compared to a neat polymer.

Recently, biochar has been used for the production of biopolymer (i.e., starch [324] and gluten [325]) composites, thus fulfilling the vision of total bio and sustainable production.

### 3.4. Other Uses of Biochar

Biochar has found plenty of applications in all the field that are traditionally occupied by carbonaceous materials such as solid fuel [326,327]. The catalysis has seen the risen of biochar in the last few years [328], with application in many processes. Lee et al. [329] developed porous catalysts from pine and maize biochar that were produced at torrefaction temperatures ranging from 300 to 380 °C for biodiesel synthesis through a pseudo-catalytic transesterification. Li et al. [330] produced biochar from hydrolyze-mixed textile waste in the temperature range of 400–700 °C, and they used it as catalyst for succinic acid production in a fibrous bed bioreactor with a yield of 8 wt.%. The amount of surface acidic groups represent a key properties for biochar catalytic activity [331]. Kastner et al. [332] used a solid acid biochar as heterogenous catalyst for the esterification of fatty acids. Biochar produced at 500 °C was tailored with sulfonic groups to reach conversion values close to 99%. Zhong et al. [333] further tailored sulfonic-decorated biohchar with alkyl groups, and they produced a highly active catalyst for biofuel production and for transesterification reactions [334].

Vidal et al. [335] developed an amino-siloxy-oxidized biochar that was able to promote the conversion of epoxy compounds and CO_2_ into glycerol carbonate.

Areeprasert et al. [336] introduced iron particles onto a biochar surface to perform catalytic reforming processes of waste electronic and electric equipment, reaching a liquid yield of up to 68 wt.%.

Furthermore, biochar could be efficiently used in redox-mediated reactions [337]. Cao et al. [338] proposed a very promising route to convert iron-enriched plant residue by using an electro-active biochar-based catalyst. The authors pyrolyzed a metal hyperaccumulator water plant and produced a biochar that contained 28 mg/g of iron. They tested the biochar that was produced in the electrocatalytic reduction of oxygenated water by using cyclic voltammetry, and they found a reduction current of up to 1.82 mA/cm^2^.

Biochar could also be used for the production of electrochemical measurement devices [339,340]. Ziegler et al. [341] use mixed softwoods that were pyrolyzed at 700 °C with a drop-casting technique for the preparation of a room temperature-relative humidity sensor. The authors clearly showed the onset of the response, with a relative humidity of 5% varying the impedance of two orders of magnitude when humidity reached 100%. Similarly, Jagdale et al. [342] used spent coffee grounds to realize a relative humidity sensor with a starting response at 20% humidity. Further studies showed the use of biochar-based materials for the detection of ions (i.e., lead [343], copper [344], and zinc [345]) at concentrations of nmol/L and for organic materials in mmol/L concentrations [346,347]. Several authors have described the use of biochar-derived materials for biosensing. Kalinke et al. [348] pyrolyzed nitric acid-treated castor oil cake at 400 °C and tailored it with Ni(OH)_2_/NiOOH for the realization of non-enzymatic glucose electrode. Alternatively, Martins et al. [349] developed an immunoassay for hantavirus detection that was based on a biochar platform with a range of work from 5 ng/mL to 1.0 µg/mL.

Biochar has also been used in biological procedures. Huang et al. [350] pyrolyzed rosin waste and tailored it with silver nanoparticles for anti-bacterial use. The use of biochar as additive in bioprocesses was reported by Bock et al. [351]. The authors used biochars to stabilize digestors because they control ammonia formation. Duan et al. [352] use biochar to improve short chain fatty acid algae anaerobic fermentation, and they doubled the amount of biomass production after four days.

## 4. Conclusions

In this review, we have presented an updated overview of non-soil applications of biochar with a focus on more useful and unusual ones. We reported many studies on the adsorbitive capacity of ions and organic molecules, together with their biochar electrochemical properties. These properties are particularly relevant in the future perspective of clean energy production and storage. We also described, in detail, the possibility of using biochars as sound replacements for traditional fillers in both inorganic and organic composites materials. This evidence has shown the feasibility of the biochars used in a lot of sectors as solid alternatives to traditional and next-generation materials. The polyhedral nature of biochar represents a very strong advantage for spread the biochar use across material science field.

We hope that this summary of recent literature can lead to the foundation of new research which will exploit the great potential of biochar and biochar based materials.
